# Performance enhancement of the solar still using textiles and polyurethane rollers

**DOI:** 10.1038/s41598-024-55948-z

**Published:** 2024-03-03

**Authors:** Jakub Wiener, Muhammad Zaman Khan, Kaushal Shah

**Affiliations:** Department of Material Engineering, Faculty of Textile Engineering, Technical University of Liberec, Studentska 2, 46117 Liberec, Czech Republic

**Keywords:** Solar still, Water distillation, Textile, Cotton fabric, Polyurethane, Exergy analysis, Energy analysis, Cost analysis, Ecology, Environmental sciences, Hydrology

## Abstract

The acquisition of clean drinking water in regions with limited power sources has been a challenge of paramount concern. Solar stills have emerged as a popular and sustainable option for obtaining clean water in such regions. This process involves employing solar radiation to heat up water, which is then condensed to obtain potable water. The present study introduces a solar still system that is both cost-effective and energy-efficient, while simultaneously ensuring sustainability. Fabric-coated polyurethane rollers with capillary action enhance evaporation area, leading to notable performance improvements. Water vapour condensed on the cooling chamber's inclined aluminium plate and collected in the distillate chamber within the solar still. The thermal, energetic, and economic performance and productivity of the proposed model were evaluated. The fabricated solar still boasted maximum instantaneous system efficiency and exergy efficiency of approximately 62.16% and 7.67%, respectively. This system's cost-effectiveness and performance improvements are particularly noteworthy. The daily average distillate productivity of the proposed still was estimated at 1.14 L/m^2^, resulting in an annual production rate of 416.54 L/year. The estimated cost of producing 1 L of distillate was 0.023 $.

## Introduction

Water scarcity has emerged as a pressing global issue with population growth, industrialization, and pollution contributing to its severity. A mere 0.014% of the Earth's total water is available for human use, as 97.5% of all water bodies are salt water, and the remaining 2.5% is freshwater. Unfortunately, obtaining this freshwater remains challenging as much of it exists in polar ice, atmosphere, and groundwater. Consequently, approximately one-third of the world's population confronts freshwater scarcities, especially in regions with inadequate access to clean water. The absence of requisite energy sources required for water desalination only exacerbates this issue. The realm of water science and technologies encompasses a significant sector of environmental engineering, with particular emphasis on the contamination of water bodies by various sources, the technologies employed in wastewater treatment plants (WWTP), and the processes utilized in water purification and desalination units. Environmental experts ought to be mindful of the potential for water bodies to become contaminated by the novel coronavirus, particularly in regions that depend on solar desalination and solar disinfection systems. This is especially pertinent in the context of systems that are poised to operate in areas such as high altitude regions^1–3^.

Seawater desalination is an energy-intensive process that creates fresh water. There are different ways to desalinate seawater, including thermal (like multi-stage flash distillation and multi-effect distillation) and membrane processes (like reverse osmosis and Electrodialysis)^4,5^. However, the most common methods of desalination can't remove all the pure water from a salty or contaminated source and must discharge up to 50% of the input. This can harm the environment and is expensive^6,7^.

Solar stills are a simpler and more effective method for smaller units because they're low-cost. Solar water treatment is a viable solution for cleaning a variety of water sources, including seawater, groundwater, surface water, and various types of wastewater, such as pharmaceutical, industrial, and urban. There are three main types of solar-powered water desalination and purification systems: solar stills, solar water disinfection (SODIS), and humidification-dehumidification (HDH). Among these, SODIS is regarded as one of the simplest and most cost-effective methods for treating contaminated water, particularly in developing countries where biological contamination is a significant issue^8^. SODIS systems are passive, low-cost, and easy to implement, making them an appealing option for addressing the challenge of contaminated water in areas with limited resources. Solar stills could be one of the most effective ways to create fresh water^9^. Therefore, exploring eco-friendly solar desalination techniques presents a significant opportunity. Solar-powered desalination units are available in various types, such as solar still (SS)^10,11^, humidification-dehumidification (HDH)^12,13^, multi-stage flashing^14,15^, reverse osmosis (RO)^16–18^, and hybrid units^19,20^.

These present several advantages, including reduced carbon footprint, as they do not require the use of fossil fuels or produce greenhouse gases during operation. They promote environmental sustainability and contribute to mitigating the effects of climate change. In recent years, solar still systems (SS), have undergone continuous development with improvement for small levels utility^21–23^. For instance, Nijmeh et al. conducted an experiment with a single-basin solar still in Amman (Jordan) during month April and May by utilizing various absorbing materials. The materials used to enhance the absorptivity of water for solar radiation include dissolved salts, violet dye, and charcoal. Moreover, through dissolving of these materials the enhancement of still efficiency and productivity was investigated^24^. In 2017, Sharshir et al. carried out a review of various types of solar stills, analyzing their thermal performance and exergy via optimization methods and designs^25^. In the same year, Yousef et al. conducted energetic performance analysis on single slope solar still. The proposed system demonstrated a maximum exergy efficiency of 2.23%^26^.

Zanganeh et al. observed that using nano-coated materials in solar stills increased the productivity. Two condensation mechanisms were tested using two different coated materials with TiO_2_ and Si nanoparticles (NPs). The results showed a significant improvement in productivity of the solar still^27,28^.

In order to enhance the water productivity of solar still desalination, researchers have looked into the use of wick materials^29,30^. Previously, a study examined the impact of various wick materials on the water yield of solar stills (SS). Jute and black cotton fabric were utilized as wick materials in two solar stills with water depths of 1 cm and 2 cm. The obtained results showed that jute fabric increased the yield by 18.03% and 21.46% at water depths of 0.01 m and 0.02 m respectively, compared to black cotton. The solar still having jute fabric and black cotton fabric produced approximately 910 mL/m^2^ and 771 mL/m^2^ respectively, at a water depth of 1 cm, which equals 828.7 mL/m^2^ and 682.3 mL/m^2^ at a water depth of 0.02 m^31^.

Kabeel et al. studied a solar still's performance and increased its productivity by 44.82% using wick materials and a V-corrugated absorber, which were then floated in water. The study showed that the productivity of the modified system was increased by 44.82% compared to traditional ones, with a distilled water generation of 6010 mL/m^2^^32^. In a previous work, the use of different types of vertical wick materials (i.e., polyester, terry cotton, woollen fabric) in solar stills showed an increase in distillate water productivity. This phenomena is due to the capillary action which raises the rate of evaporation^33^. Kalidasa Murugavel and Srithar tested various wick materials in solar stills with a 0.5 cm water depth. They found light black cotton cloth to be the most effective wick material^34^.

Despite the benefits of traditional solar desalination systems, they are known to be costly, inefficient, and fragile. Recent research has suggested that textile-based solar desalination systems can offer a more effective and affordable solution^35,36^. By utilizing woven materials to harness solar radiation, water desalination can be achieved more efficiently and at a lower cost. Given the worldwide water scarcity, it is crucial to explore innovative solutions such as textile-based solar desalination to provide clean water to millions of people around the world^37,38^.

The process of solar distillation with the use of textiles is more effective compared to glass or metal. Textiles offer the advantage of being lightweight, flexible, and easily adaptable to the needs of different communities. Among the various materials for solar distillation systems, polystyrene nonwoven is a highly promising choice due to its absorbent, lightweight, and cost-effective properties. Researchers are currently investigating the use of polystyrene nonwoven in solar distillation to enhance system performance and lower costs. This material is highly resistant to UV rays and heat, resulting in a more durable and efficient performance in harsh solar distillation systems, unlike other textiles such as polyester (PET) and polyamide. In fact, polystyrene nonwoven has outperformed PET and polyamide textile systems in solar distillation, producing more distilled water by absorbing more distillation by-product water vapor. Textile-based solar distillation is being researched for desalination and wastewater treatment plants, offering the potential to provide clean water to underserved communities while improving system efficiency and sustainability^22,23,39^.

Textile solar stills represent a cost-effective and versatile solution for solar distillation due to their lightweight design, thermal insulation, and light transmissivity properties. Despite the numerous benefits they offer, textile-based solar distillation faces several challenges, such as ensuring the textile materials can withstand extreme climatic conditions, such as high temperatures and UV radiation, without deteriorating or losing their effectiveness. Textile materials are flexible and can be customized to meet specific needs, including durability, thermal insulation, and light transmission. It has been reported in previous studies that the efficiency of a solar still (SS) largely depends on the surface and evaporation rate, and that the use of various wick materials can improve the evaporation rate. In particular, textile-based materials have demonstrated promise in enhancing the evaporation rate and boosting the output of traditional single-slope solar stills. However, there is currently no research on the efficiency and performance of solar still (SS) via a horizontal wick roller technique and cooling chamber. This study aims to address this gap in the literature by utilizing a fabric-coated polyurethane-based roller wick as a new type of efficient and safe absorber for solar still systems. The experimental portion of this study focuses on two critical aspects: roller cover fabric selection and condensation mechanism. These factors are crucial for determining the overall performance and efficacy of the experimental setup. Polyurethane foam rollers have unique properties that improve solar still functionality due to their low thermal conductivity.

The present study aimed to enhance the efficiency of solar stills by improving the evaporation and condensation processes. Specifically, the study employed polyurethane foam rollers coated with black cotton fabric, which led to a higher rate of wicking and productivity. Additionally, the cooling chamber was installed to increase the rate of condensation. The modifications were designed to maximize the evaporation rate and to prevent the condensation of water on the slope of the solar still. Through the use of theoretical analysis, the efficiency of the proposed solar still was simulated and predicted. The basin uses black textile rollers for evaporation, offering advantages over traditional basins. The thin layer of water on the rollers has minimal thermal inertia, allowing a quicker start-up. The localized heat increases surface water temperature, leading to faster evaporation. The floating roller allows for flexible water depth, preventing dry-out conditions while maintaining performance. The results of the study revealed that the modifications led to a significant improvement in performance at a relatively low cost.

## Experimental setup and process

**Figure 1:**
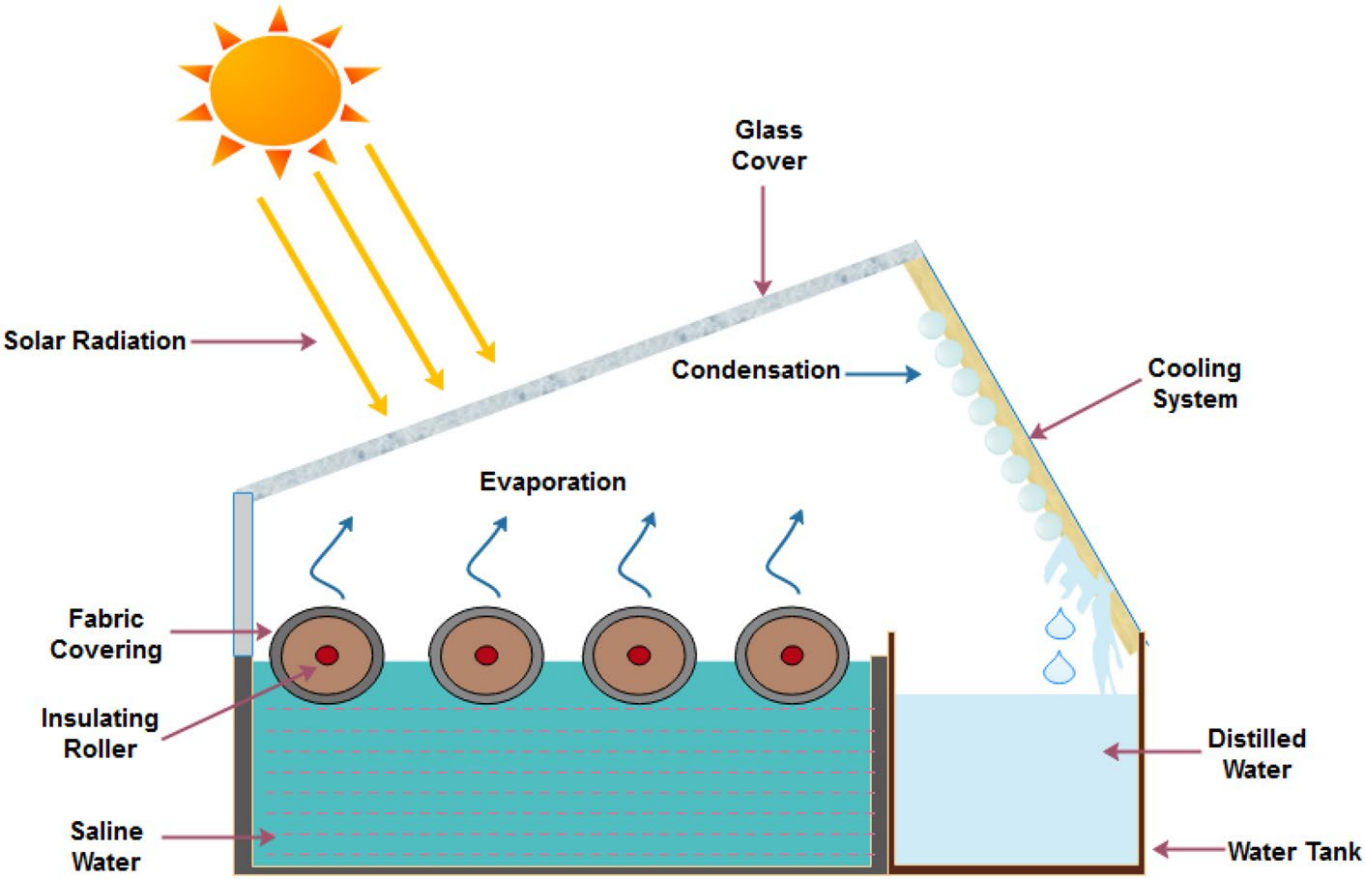
Schematic diagram of solar still experimental setup.

**Figure 2:**
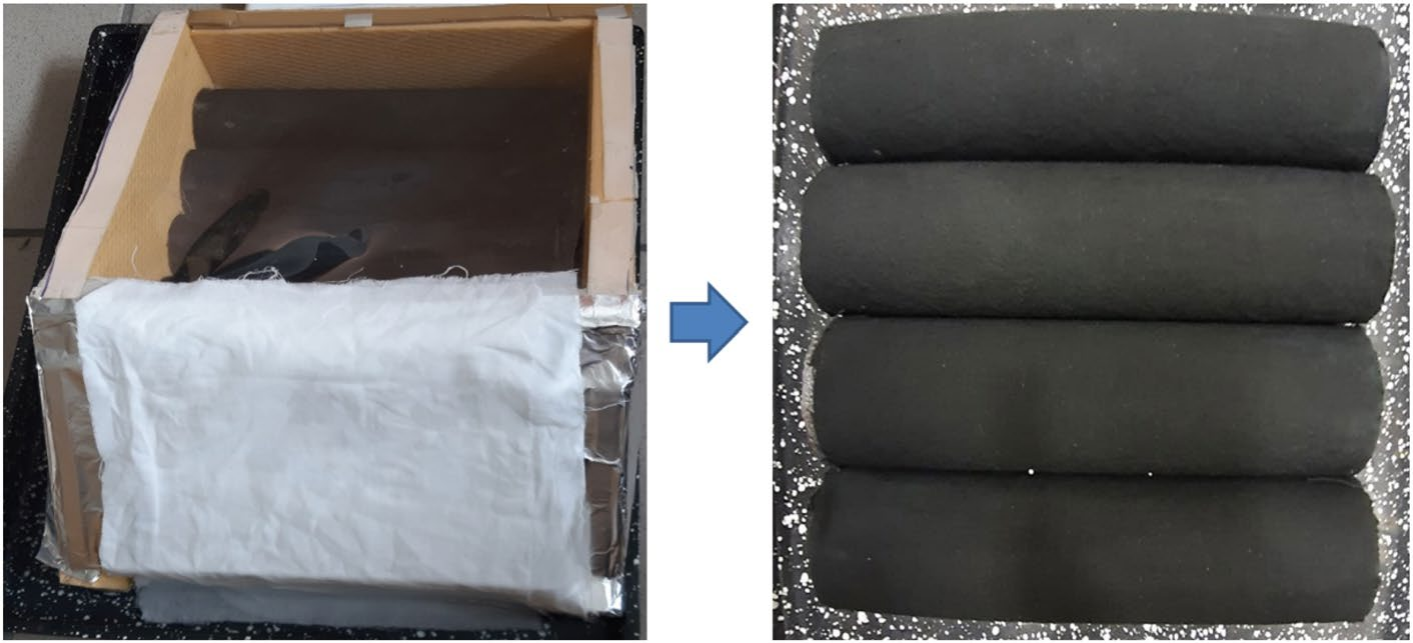
Front view of the experimental set up and black textile rollers.

**Figure 3:**
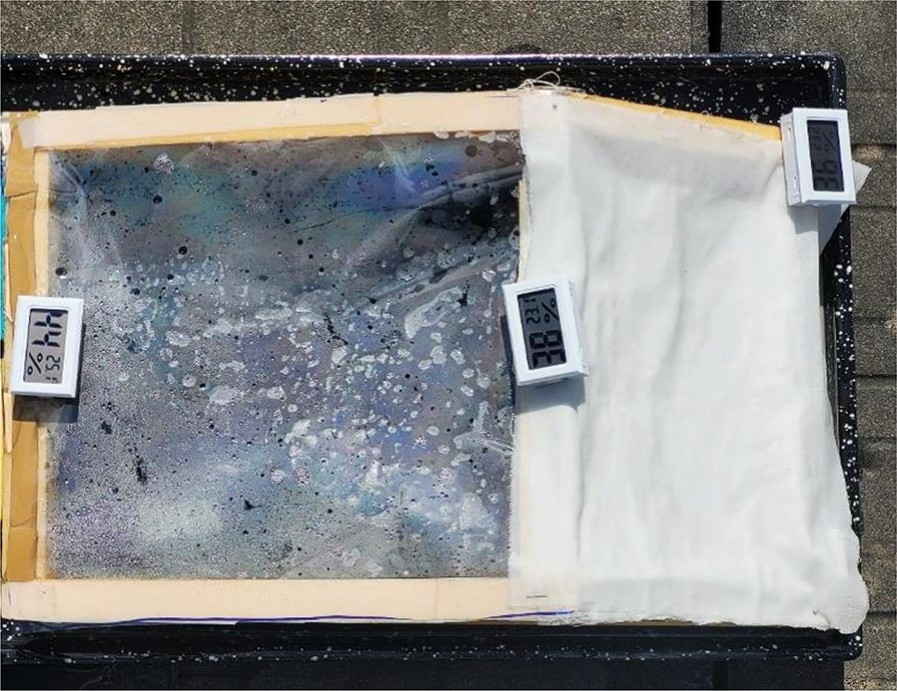
Top view of the installed set up during experiment.

In this research work, polyurethane foam rollers covered with textile fabrics was utilized as a floating absorber in solar stills to raise the level of surface area available for evaporation. In addition, in order to reduce the amount of heat lost and to speed up the pace of evaporation a textile based rollers installed solar still was designed and simulated to study the productivity of a solar still. Figure 1 shows the schematic diagram of the fabricated solar still system. First, the chamber is covered with an angled transparent cover that lets solar heat in and creates vapor from polluted water due to partial pressure difference. A transparent lid with 30° tilt was used to cover the solar still. The solar still basin having four rollers as wick materials having total area of 0.16 m^2^ and dimensions of 0.4 m × 0.4 m were used for maximum absorption of solar radiations. The condensed water vapours accumulated on the inclined inner surface of the aluminium plate of the cooling chamber was flowing and collected into the distillate chamber. The aluminium plate was kept cool using a wet cotton fabric. Silicone glue was used to fix all the parts together. The cooling chamber was covered by wet cotton fabric to enhance cooling chamber performance. Plain weave cotton fabric ventilates, prevents heat build-up and protects the cooling chamber from sunlight. This simple but effective method will improve the solar distillation system without additional energy or complicated processes. The water basin was coated with black paint to increase its absorption ability. The schematic representation and photograph of solar still setup are shown in Fig. 1. Figures 2 and 3 showed the front and top views of experimental setup in real working conditions.

### Dyeing process

**Figure 4:**
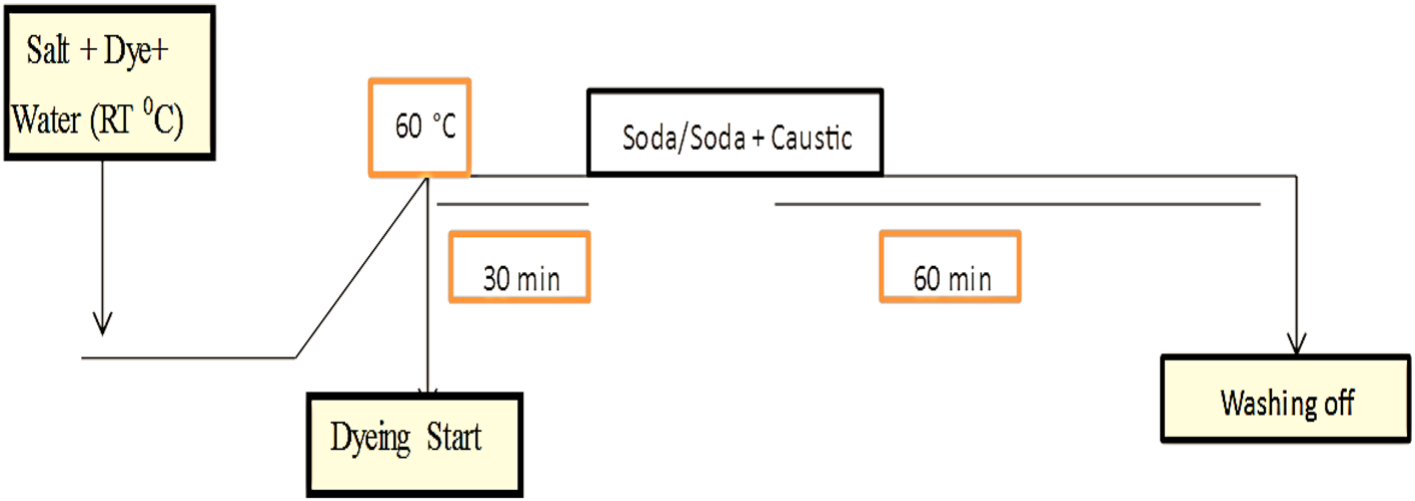
The schematic of the cotton fabric dyeing using Direct Black 22 dye.

Using a textile material that can absorb the most solar radiation is essential for maximizing the thermal efficiency and production rate of a solar apparatus used for solar water distillation. Fabrics dyed with black, which are known for high absorptivity of solar radiation, may be used for solar water distillation. As high amount of solar energy is absorbed by the black dye and transferred to the water, the thermal efficiency of process is improved when employed in a solar apparatus. This raises the water's temperature, which in turn increases the pace at which distilled water may be produced. Since the black dye is less susceptible to fading or degradation from exposure to solar radiation over time, its usage may also aid to enhance the endurance of the textile material. The following dye saturnová čerň A was used for the dyeing process. The schematic procedure for dyeing the cotton is same for the both dyes as shown in Fig. 4.

### Characterization techniques

In order to evaluate the performance and viability of different systems and processes, testing is necessary. To assess the efficiency, effectiveness, and economic feasibility of the solar distillation apparatus in the context of water distillation using black dye textile material, several tests are conducted. This study employs various testing and evaluation techniques, such as the evaporation rate, wicking rate, Colorimetric (CIELAB) analysis, thermal efficiency, production rate, and economic viability. These tests provide valuable information about the system's evaporation rate, water-wicking capacity, distillation rate, energy efficiency, production capacity, and overall economic feasibility. By analyzing these factors, we can determine the system's efficiency and identify areas for potential improvement.

### Wicking test

To conduct a wicking test, a small rectangular sample of the fabric is immersed vertically into a container filled with water or any other liquid, with a marked line indicating the starting point of the liquid level. This line also shows the initial height of the liquid level. After a set amount of time, usually ranging from 10 to 30 s, the fabric is taken out, and the level to which the liquid has risen while being absorbed is measured. This measurement indicates the wicking capacity of the fabric, which refers to its ability to effectively absorb and move liquids at a certain rate.

### Evaporation test

The rate of evaporation may vary for different textiles depending on a number of parameters including the kind of weave, the thickness, and the composition. IR heater having power of 800 watts was used for evaporation test. The rate of evaporation was found to vary depending on the kind of textile material used during testing that was carried out at 54 cm using polyurethane rollers that were covered with woven, knitted, and non-woven textile structures. During the experiment, the room temperature was maintained at 22.8 °C, and the humidity was maintained at 28%; both factors had an impact on the evaporation rates. It was owing to IR Light that the temperature on the testing side was 40 °C, while the humidity was 58%.

### Colour strength and Colorimetric (CIELAB) analysis

The colour strength, as measured by K/S value, and colorimetric values, comprising L*, a* and b*, were quantified using a spectrophotometer for colour measurement (FOTOCHROM 3), with illuminate D-65 and 10 observers. The K/S value was derived using the Kebelka-Munk Eq. (1).

1 $$K/S=\frac{1-{R}^{2}}{2R}$$

Here, R indicates the percentage reflectance, K the absorption coefficient, and S the scattering coefficient.

### Thermal and exergy efficiency

The thermal efficiency of a solar still can be determined by calculating the ratio of water generation to the inlet solar energy. Similarly, the hourly thermal efficiency of the solar still can be computed. It measures the system's thermal efficiency by using energy for freshwater generation divided by the total input radiation rate. An exergy analysis is a useful tool for determining the amount of work that a system can produce while also revealing any energy losses or inefficiencies. In other words, the exergy efficiency of a system is the ratio of useful exergy output to total exergy input, taking into account any losses due to inefficiencies.

### Production and economic viability

Measuring the production rate is crucial to determine the efficiency and effectiveness of a solar still. It indicates the amount of water distilled by the solar still per hour in liters (L/h). The profitability of a solar still water distillation system can be evaluated by considering various factors. These include the cost of the apparatus, operating expenses, water production rate, water demand, and water cost. In order to establish the economic feasibility of the system, it is necessary to compare the fixed costs and operating expenses against the quantity of water generated and the water demand. If the costs are lower than the cost of purchasing water, the system may prove to be economically viable. Conversely, if the costs exceed the cost of water, the system may not be a feasible option. Therefore, it is crucial to conduct a thorough cost–benefit analysis before investing in a solar still water distillation system, in order to make an informed decision.

## Results and discussion

**Figure 5:**
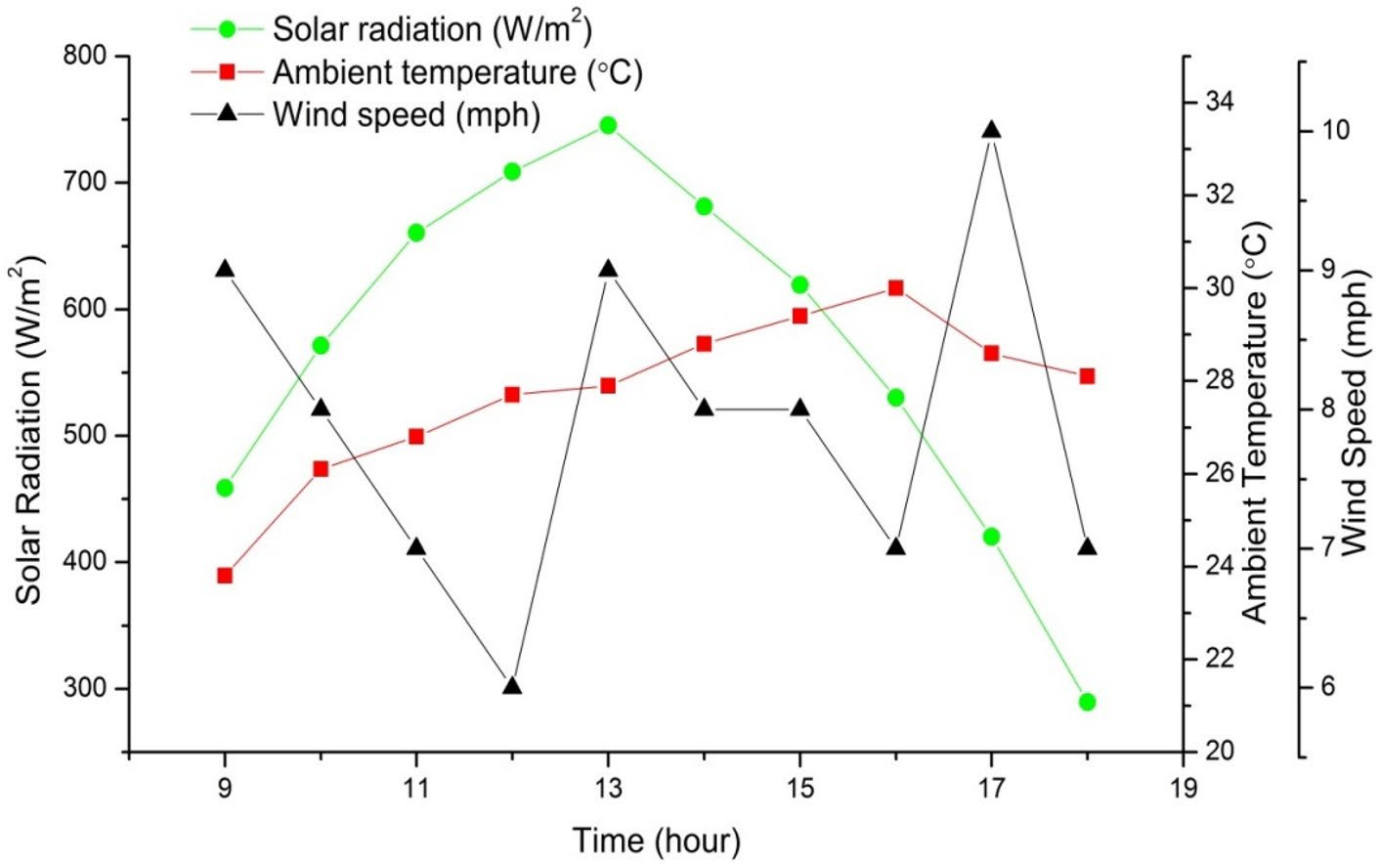
Variations of solar intensity, ambient temperature and wind speed.

To efficiently and practically evaluate various systems and processes, observation and calculation are two vital tools. In the case of a solar apparatus that uses black dye textile material for water distillation, there are several crucial aspects that must be observed and calculated to assess the apparatus's efficiency, effectiveness, and economic viability. To evaluate the solar apparatus, you need to conduct the evaporation test, which measures the rate at which water evaporates from the apparatus. Additionally, the wicking test evaluates the textile material's ability to draw water through capillary action. The rate at which the solar apparatus for water distillation operates determines the speed at which distilled water is produced. The thermal efficiency of the system gauges how effectively heat is transferred within the system, and the production rate evaluates the amount of distilled water generated over a specific time period. Finally, economic feasibility calculations consider the cost-effectiveness and feasibility of implementing the system. By carefully observing and calculating these factors, you can gain valuable insights into the system's performance, allowing you to identify areas that require improvement. Figure 5 illustrates the changes in ambient temperature, solar radiation, and wind velocity over time for one of the typical test days. The figure displays the hourly variation in wind speed and ambient temperature throughout the day. It is evident from the figure that the solar radiation increases steadily and reaches its maximum value at midday, after which it starts to decrease.

### Wicking and evaporation analysis of the textile materials

**Figure 6:**
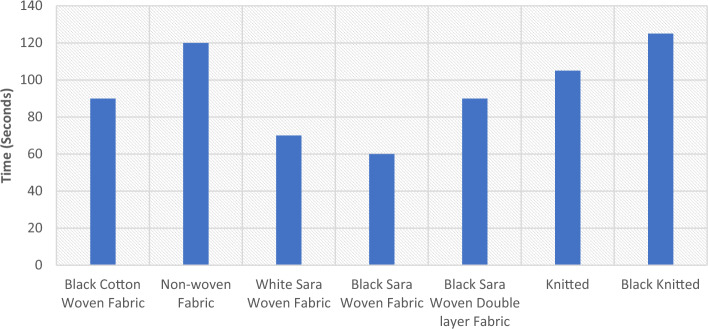
Water saturation by wicking of various textile structures.

A wicking test was performed on different types of textiles, including woven, knitted, and nonwoven fabrics, to evaluate their effectiveness in a solar device (Fig. 6). A small rectangular sample of each fabric was vertically inserted into a dish containing water, which served as a test chamber. The liquid level was marked, indicating the starting and ending points. Measuring the height to which the liquid had risen on each fabric sample provided an indication of its wicking capacity. The test also allowed for the observation of how each fabric behaves when it comes into contact with liquid, such as the rate of liquid absorption and release, which affects the rate of evaporation. These factors play an essential role in determining the suitability of a fabric for use in a solar device. A record of the observations was made on a table, which was then evaluated to determine the most appropriate fabric structure for a solar device. The knitted and nonwoven fabrics demonstrated a high level of wicking ability. The test results provide critical information on the performance of materials under different conditions. The wicking and drying properties of fabrics are significantly influenced by raw material and fabric structure selection. In general, knitted fabric structures possess good wicking and moisture absorption properties^40,41^. Wetting, wicking and evaporation are critical characteristics that affect moisture management of the fibrous structures^42^. Liquid can move through a fabric by flowing through the spaces between the yarns and fibers that make it up, which is primarily due to capillary action. Therefore, how much space there is both between and within the yarns of a fabric is expected to affect how well liquid can travel through it^43^.

**Figure 7:**
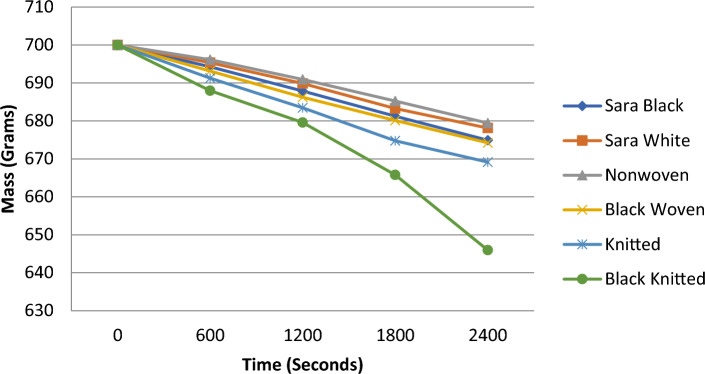
Evaporation analysis of the various textile structures.

The present study aimed to evaluate the performance of polyurethane foam rollers coated with woven, knitted, and nonwoven textiles through an evaporation test. The purpose of this evaluation was to provide essential insights into the overall effectiveness of these rollers. The evaporation test results, illustrated in Fig. 7, demonstrate the mass reduction of the polyurethane foam rollers over different time intervals 0, 600, 1200, 1800 and 2400 s. The black knitted fabric-covered roller exhibited the highest evaporation rate, with the mass of the roller reducing to below 650 g after 2400 s. The normal knitted fabric also showed better evaporation of water than the woven and non-woven structures. To ensure accuracy and reliability, various environmental parameters such as temperature, humidity, and sun exposure were carefully monitored and controlled for each roller.

The study aimed to provide empirical data to support the effectiveness of various textile materials in a solar still. The results indicate that the fabric structure is critical for achieving effective liquid management properties. The knitted fabric structure is found to be superior to woven and non-woven structures in terms of wicking and quicker evaporation properties. These results have significant implications for the design of solar still and can pave the way for the development of more efficient solar still. Overall, this study underscores the importance of careful evaluation of textile materials in the design of solar still. The results can be used as a basis for further research on the development of advanced textile materials that can enhance the effectiveness of solar stills^44^. There are various studies on the liquid management properties of fabric, which rely on fiber, yarn properties, and textile structure^45–48^.

We discovered that textile material-coated polyurethane foam rollers are the most effective material for the solar water distillation system after conducting an evaporation test. This system is sustainable, portable, and easy to use, making it suitable for various applications. By using textile materials, the system provides a simple and efficient approach to water purification, without the need for external energy sources or human intervention.

Airflow and evaporation are slow in solar devices without knitted or nonwoven materials, reducing efficiency and performance. Woven fabrics are durable and breathable. Woven fabrics are rip-resistant. Over-under weaving creates a tight, flat fabric. Woven textiles are stable and don't stretch. Solar equipment requires fabric to retain its shape over time. Woven fabrics are more breathable. Evaporation requires airflow, which may benefit a solar device. Woven fabrics are more durable because they resist ripping. Covering the polyurethane foam roller with a black dye cotton fabric improves water collection, energy efficiency, durability, and environmental resistance, which may increase solar distillation systems' effectiveness. Solar distillation systems can be cheaper for communities that need clean water due to low-cost and widely available materials. Solar distillation systems with polyurethane foam rollers and cotton fabric covers need more research to determine their optimal design and performance in different environmental conditions.

### Colorimetric analysis

**Figure 8:**
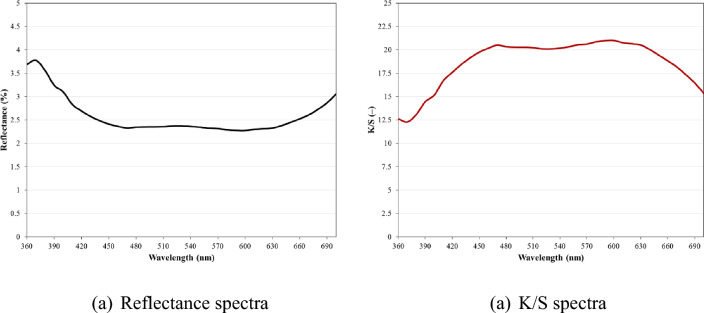
Colorimetric analysis of the dyed fabric.

The colorimetric analysis of black-dyed fabric was conducted to evaluate various parameters. The K/S value for the dyed cotton fabric was found to be 18.67, indicating a significant change in the fabric's colour from light to dark. The L* value of the dyed fabric sample was determined to be 17.17. The a* value for the black coloured fabric was positive (0.25) and b* values for the black coloured fabric was negative (− 0.88), suggesting reddish and bluish shades, respectively. The colour strength of dyed fabric is expressed using K/S values, which are calculated based on the reflectance values. A lower reflectance percentage of 2.59 indicate that the incident light is absorbed more^49–51^. Figure 8 displays the Reflectance (%) and K/S values of black-dyed fabric.

## Theoretical consideration

### Energy analysis

The thermal efficiency of a solar still is calculated as the ratio of water produced to the energy input from the sun. This metric is vital for evaluating the effectiveness of solar stills, as it provides an indication of how efficiently they convert solar energy into clean water. As such, it is essential to accurately measure the energy input and water output to determine the thermal efficiency of a solar still. The hourly thermal efficiency is defined as^52–54^:

2 $$\eta =\frac{{M}_{evp}\times {LV}_{evp}}{\left({I}_{ss}\times {A}_{abs}\right)\times 3600}\times 100$$

where M_evp_, LV_evp_, I_ss_, and A_abs_ indicate the rate of yield, latent heat of vaporization of water, solar intensity, and the area of basin, respectively.

The solar stills that were created have been evaluated for their effectiveness in converting input energy from a light source to usable heat energy for water distillation. The thermal efficiency test considered various factors, including the latent heat of vaporization, which is the heat required for liquid-to-gas transformation at a constant temperature. This test revealed that the fabricated solar stills have a maximum instantaneous system thermal efficiency of approximately 62.16%. The thermal efficiency of the solar stills is subject to environmental factors such as temperature and humidity, which can affect their performance. The area of the involved cylinders plays a crucial role in the energy conversion for water distillation, which also influences the thermal efficiency of the solar device. Overall, the thermal efficiency test provides valuable insights into the performance of the solar stills. These insights can help improve the design and functionality of solar equipment, making it more effective and efficient in converting energy from light sources to heat energy.

### Exergy analysis

A thorough analysis of the exergy efficiency of a solar still indicates how effectively it converts the input energy from the sun into evaporation energy for water distillation. The outcome of the analysis is determined by calculating the exergy efficiency rating of the solar still. The rating is an indicator of the effectiveness of the solar still in converting the input energy into heat for the distillation of water. Higher exergy efficiency ratings indicate that the solar still can more efficiently transform the input exergy into heat for freshwater generation.

The exergy efficiency is calculated as the ratio of the energy used for freshwater generation to the total input radiation rate. This means that the more energy the solar still uses for freshwater generation, the higher the efficiency rating will be. The analysis of exergy efficiency is crucial because it helps to determine the effectiveness of the solar still in distilling water from the sun's energy. The exergy efficiency of the system can be defined as^10,55^:

3 $${\eta }_{EXG}=\frac{{EXG}_{evap}}{{EXG}_{input}}$$

In the above equation, η_EXG_, EXG_evap_, and EXG_input_ are exergy efficiency of the system, exergy of evaporation of the system, and exergy input of the system, respectively.

The exergy input was determined as following:

4 $${EXG}_{Input}=\frac{{A}_{sample}}{{A}_{total}}\times P$$

where EXG_Input_ is the amount of exergy used to heat the sample in joule per second, A_Sample_ is the area of sample in m^2^, A_Total_ is the total area of the system in m^2^, and P is the applied power, which is 800 Watt.

The EXG_evap_ is the exergy of evaporation of the system also called exergy output of the system is calculated by^10,56^:

5 $${EXG}_{evap}=\frac{{M}_{evap}\times {LV}_{evp}}{3600}\times \left[1-\frac{{T}_{a}}{{T}_{w}}\right]$$

where LV_evp_ is the latent heat of vaporization of water in (J/g), M_evap_ is the mass of water evaporated in grams, T_a_, is ambient temperature, and T_w_ is the water temperature, respectively. The latent heat of vaporization is the amount of exergy required to vaporize one gram of water. Therefore, the latent heat of vaporization is approximately 2260 J/g.

Exergy efficiency is an important factor to consider in any system. The efficiency of a system can be improved by reducing exergy waste and increasing exergy output. In this regard, a modified still was developed to enhance exergy efficiency. The still was equipped with black-coloured textile-covered rollers, which increased the surface-to-volume ratio. This increased the absorption of incident radiation and improved the evaporation surface, leading to higher production of distilled water. As a result, the exergy efficiency of the modified system was significantly improved and reached a maximum of 7.67%.

It is worth noting that in a previous study, researchers explored the performance of the solar still via utilizing reticular porous membrane. This modification was found to significantly enhance exergy efficiency, achieving a maximum exergy efficiency of 7.33%. The study concluded that the insertion of a reticular porous membrane could potentially improve the performance of solar stills and enhance exergy efficiency^57^.

### Production rate

**Figure 9:**
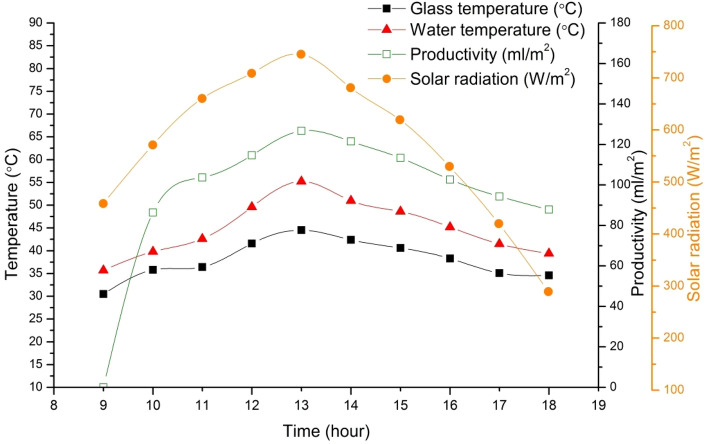
Variation of solar intensity with hourly productivity and temperatures of SS.

Figure 9 shows the relationship between the glass and water temperature, hourly productivity, and solar radiation for the solar still that was constructed. In the early hours of the experiment, the water temperature increases gradually as solar radiation increases. However, after 14:00, there is a sharp decrease in solar radiation, but the water temperature decreases slowly, which is because water has a high latent heat. The slope of the water temperature is steep in the early hours of the experiment due to the increase in solar intensity. The temperature difference between the water and glass is lower in the early hours of the experiment (until 11:00) than it is between 11:00 and 18:00, resulting in a higher rate of condensation. Therefore, the efficiency of the solar still is directly related to solar intensity, which is stronger in high-altitude areas.

The production rate indicates the volume of water distilled by the device in liters per hour (L/h), enabling assessment of optimal operation and identification of potential enhancements. Fresh water productivity (p) of the solar still is calculated as follows:

6 $$Productivity (p) =\frac{Water \;produced ({W}_{Ph})}{Time (t)}$$

Calculating the production rate of a solar water distillation device is an essential step in assessing its efficiency and productivity. To do this, the total water quantity is divided by the test duration, considering variables such as solar intensity, initial water temperature, and environmental conditions. The productivity rate can then be calculated by converting the amount of water distilled after 1 h from grams to liters, resulting in a maximum productivity rate of 0.126 L/m^2^. It is important to note that in many studies, the findings from the summer season are applied to other seasons as well. However, this may not be entirely accurate as the system can function for up to 12 h during summer, whereas it may operate for less than 9 h in autumn and spring. Based on the calculations, the device's exergy efficiency was found to be 7.67%, with an area of 0.16 m^2^. These values were utilized to calculate the production rate based on 12 h operation, which was determined to be 416.54 L/year or an average of 1.14 L/day. Our results are in accordance with the previous finding of the studies^58,59^. Accurate measurement of the production rate is crucial, considering factors such as light intensity and environmental conditions, to assess optimal operation and potential modifications. The calculated average production rate of 1.14 L/day highlights the device's potential for effective water distillation. Further research can focus on optimizing parameters to enhance efficiency and production rate. It is essential to note that the production rate serves as a crucial indicator of the device's efficiency and productivity in solar water distillation. Therefore, accurate measurement and assessment are imperative for achieving optimal performance.

### Cost analysis

Solar still distillation systems on textile-based materials are efficient source of renewable energy. The economic analysis of such systems is a critical factor in determining their practicality and feasibility. To perform an economic analysis, several variables are taken into account, including the production cost, the value of manufacturing and the water output. The cost of productivity refers to the expenses associated with distilled water production. The manufacturing value is the total cost of materials, labour, and other expenses involved in building and operating the system. The value of the water output is the price that can be charged for the distilled water produced by the system. To determine the price of one liter of distilled water, the annual cost of the system is divided by the annual water production rate. Overall, economic analysis plays a crucial role in evaluating various solar energy systems, including solar still distillation systems that use textile-based materials. By understanding the economics of such systems, stakeholders can make informed decisions about their feasibility, practicality, and long-term sustainability.

Solar stills are a cost-effective means of producing freshwater and have a comparatively shorter payback period. In calculating the fixed annual cost of the still, with due consideration of a 12% interest rate and a 10-year lifespan, the necessary inputs are the capital recovery factor and capital cost. The capital recovery factor of a solar still can be obtained using the equations presented below^60^.

7 $$FAC=CRF\times CC$$

8 $$CRF=\frac{i{(1+i)}^{n}}{{(1+i)}^{n}-1}$$

where CC represents the capital cost. Annual salvage value (ASV) can be calculated using the sinking fund factor and salvage value, which is 20% of the capital cost^60^:

9 $$ASV=SSF\times S$$

10 $$S=0.2\times CC$$

11 $$SFF=\frac{i}{{(1+i)}^{n}-1}$$

where S represents the salvage value of the system. The annual maintenance cost is 10% of its fixed annual cost and is achieved as follow^60^.

12 $$AMC=0.10\times FAC$$

13 $$UAC=FAC+AMC-ASV$$

where UAC is the uniform annual cost of the system. The cost per litre of distillate and the payback period are calculated. This calculation provides a clear understanding of the price of distilled water and how it relates to the cost of production and is given as follow^56,61^:

14 $$CPL=\frac{UAC}{\left({MP}_{aveg}\right)yearly}$$

Different capacity-increasing methods can affect production cost per liter (CPL)^7,62^. The productivity of solar stills can be improved by using textile-based materials in various solar still designs. Table 1 presents the cost analysis of the distillate water cost per liter (CPL).

**Table 1:** Economic evaluation of designed solar still.

n (years)	i (%)	FAC ($)	S ($)	AMC ($)	UAC ($)	Annual yield (L/m^2^)	CPL($/L)
10	12	8.48	9.43	0.85	9.66	416.54	0.023

Table 2 presents the comparison of cost per litre (CPL) of distilled water on solar stills using various technologies. In present study, a designed textile-based solar still system was found to have a lower cost per liter (CPL) of 0.023 $. In previous studies, the lowest CPL of 0.0051 $/L was obtained using a single slope solar desalination system with bubble wrap^29^. Previous research endeavors have examined the cost per liter (CPL) of four different types of solar stills. Results indicate that the CPL of conventional solar stills, porous surface absorber solar stills, PCM porous surface absorber solar stills, and nano-enhanced PCM-porous surface absorber solar stills were 0.121, 0.107, 0.106, and 0.098 $/L, respectively^63^. Previously, enhanced solar desalination technique utilizing 2 cm-sized stones resulted in a production cost of 0.017 $/L/m^2^, whereas the traditional method cost was 0.02 $/L/m^2^. Incorporating a porous fine black stone absorber led to a reduction in water production expenses by 8.2–17.53% when compared to the conventional type^64^. In a previous work, CPL for distilled water production using modified solar desalination with sponge rubber and conventional stills was found to be 0.0095 and 0.0108 $/L/m^2^^57^.

**Table 2:** The economic analysis of the various solar stills.

Type of system	References	CPL ($/L)
Single slope using conch shells (Biomaterial)	^55^	0.027
Single slope using gravel coarse aggregate sensible heat storage	^65^	0.061
Double slope using glass cover cooling	^66^	0.243
Tubular shape consist of vibrator wire mesh	^67^	0.014
Textile roller based solar still	This study	**0.023**

Significant values are in bold.

The cost of fixed expenses, such as equipment acquisition, installation fees, and initial setup expenses, are evaluated in conjunction with operational costs. This study examined the cost of materials used in the system and calculates the annual cost based on the provided values. The availability and increasing cost of freshwater worldwide highlight the necessity for sustainable and cost-effective solutions. Solar water distillation systems offer a promising approach, leveraging textile technology to create lightweight and portable devices. The present study emphasizes the economic attractiveness and ecological sustainability of solar devices in producing high-quality freshwater at a minimal cost. The calculated cost of freshwater production is presented, emphasizing the economic viability and potential for communities in need of reliable access to clean water.

In the face of rising costs and scarcity of freshwater, sustainable and cost-effective solutions are becoming increasingly necessary. Solar water distillation systems are a viable option for communities seeking dependable access to clean water. The cost analysis demonstrates the economic attractiveness of solar devices, which utilize textile technology to create efficient and portable systems. These systems leverage the power of the sun to produce high-quality freshwater at a minimal cost. This cost-effective and environmentally friendly approach addresses the challenges posed by traditional freshwater generation methods. Further research can focus on optimizing system design and exploring implementation strategies in water-stressed regions.

## Conclusion

In present research work, the black dyed fabric coated PU rollers were inserted in the basin of the still and influence of the fabric coated rollers on the performance and efficiecy of the solar still was experimentally investigated. The solar device demonstrated that black dyed fabric was the most efficient in absorbing and evaporating water. Moreover, the thermal and exergy efficiencies of the fabricated solar still (SS) were analysed. Moreover, economic analysis was carried out for cost estimation of distilled water.

Based on the results obtained from the experimental work, the main findings of this study are concluded as follows:

1. The efficiency improves by deploying the fabric coated rollers inside the basin due to the fast wicking and evaporation processes.
2. The maximum efficiency of the fabricated solar still reached about 62.16%.
3. The maximum exergy efficiency obtained from the fabricated SS was 7.67%.
4. The average distillate productivity of the proposed still during the 12 h time was about 1.14 L/m^2^.
5. The cost analysis showed that the estimated CPL of the distilled water production from the fabricated solar still is 0.023 $.
6. The system demonstrated a significant improvement in thermal efficiency and water yield compared to similar systems, at a lower cost.

In summary, the study demonstrated that the use of black fabric-coated polyurethane rollers and a cooling chamber can effectively enhance the efficiency of solar stills. The improvements in the evaporation and condensation processes led to increased productivity and reduced cost. The study's findings may have significant implications for the development of more efficient and cost-effective solar stills in the future.

## Future work

- The performance of solar stills can be improved by using optimized wick materials with various shapes and sizes, which require further investigation.
- Further research is also needed on the use of various nanomaterials and phase change materials coating on textiles to enhance the absorption of solar radiation.
- Investigating the use of different nanostructures and biomaterials as energy storage media could also improve the performance of solar stills.
- Additionally, the productivity of solar desalination could be enhanced by using different micro/nanofiber-based textile structures, which is an area for future research.

## Data Availability

All data generated or analysed during this study are included in this published article.

## References

[1] Masoud S, Norozpour F, Elsheikh AH, Kabeel AE (2023). Solar desalination/purification (solar stills, humidification-dehumidification, solar disinfection) in high altitude during COVID19: Insights of gastrointestinal manifestations and systems’ mechanism. J. Hazard. Mater. Adv..

[2] Masoud S, Javadi D, Rahbar A, Majidniya M (2019). Experimental assessment on passive solar distillation system on Mount Tochal at the height of 3964 m: study at high altitude. Desalination.

[3] Masoud S, Javadi D, Rahbar A, Majidniya M, Salimi M (2020). Experimental investigation at a summit above 13,000 ft on active solar still water purification powered by photovoltaic: a comparative study. Desalination.

[4] Taghavian, H., Cernik, M. & Vorak, L. D. Superhydrophilic surface modification of PTFE hollow-fiber membrane with advanced biofouling properties for water purification. In *Proceedings 14th International Conference on Nanomaterials—Research & Application* 91–96 (2022).

[5] Taghavian H, Černík M, Dvořák L (2023). Advanced (bio) fouling resistant surface modification of PTFE hollow—fiber membranes for water treatment. Sci. Rep..

[6] Shadi M, Abujazar S, Fatihah S, Anwar I, Kabeel AE (2018). Productivity modelling of a developed inclined stepped solar still system based on actual performance and using a cascaded forward neural network model. J. Clean. Prod..

[7] Kandeal AW, El-shafai NM, Abdo MR, Kumar A, El-mehasseb IM, Maher I, Rashad M, Kabeel AE, Yang N, Sharshir SW (2021). Improved thermo-economic performance of solar desalination via copper chips, nanofluid, and nano-based phase change material. Sol. Energy.

[8] Masoud S, Rahbar A, Koleini MH, Javadi YD, Afrand M (2020). First approach on nano fluid-based solar still in high altitude for water desalination and solar water disinfection (SODIS). Desalination.

[9] Masoud S, Rahbar A, Koleini MH, Aberoumand S, Afrand M (2020). A renewable energy-driven thermoelectric-utilized solar still with external condenser loaded by silver/nano fluid for simultaneously water disinfection and desalination. Desalination.

[10] Tiwari GN, Dimri V, Chel A (2009). Parametric study of an active and passive solar distillation system: Energy and exergy analysis. Desalination.

[11] Alwan NT, Ahmed AS, Majeed MH, Shcheklein SE, Yaqoob SJ, Nayyar A, Nam Y, Abouhawwash M (2022). Enhancement of the evaporation and condensation processes of a solar still with an ultrasound cotton tent and a thermoelectric cooling chamber. Electronics.

[12] He WF, Huang L, Xia JR, Zhu WP, Han D, Wu YK (2017). Parametric analysis of a humidi fi cation dehumidi fi cation desalination system using a direct-contact dehumidifier. Int. J. Therm. Sci..

[13] Hamed MH, Kabeel AE, Omara ZM, Sharshir SW (2015). Mathematical and experimental investigation of a solar humidification—dehumidi fi cation desalination unit. Desalination.

[14] Alsehli M, Choi J, Aljuhan M (2017). A novel design for a solar powered multistage flash desalination. Sol. Energy.

[15] Kaheal MM, Chiasson A, Alsehli M (2023). Component-based, dynamic simulation of a novel once through multistage flash (MSF-OT) solar thermal desalination plant. Desalination.

[16] Shalaby SM, Sharshir SW, Kabeel AE, Kandeal AW, Abosheiasha F, Abdelgaied M, Hamed MH, Yang N (2022). Reverse osmosis desalination systems powered by solar energy: Preheating techniques and brine disposal challenges—A detailed review. Energy Convers. Manag..

[17] Ali E (2022). Optimal control of a reverse osmosis plant for brackish water desalination driven by intermittent wind power. Membranes Basel.

[18] Lacroix C, Guillaume B, Perier-muzet M, Stitou D (2022). Feasibility analysis of a thermo-hydraulic process for reverse osmosis desalination: Experimental approach. Appl. Therm. Eng..

[19] Sharshir SW, Hamada MA, Kandeal AW, El-said EMS, Mimi A, Rashad M, Abdelaziz GB (2021). Augmented performance of tubular solar still integrated with cost-effective nano-based mushrooms. Sol. Energy.

[20] Sharshir SW, Peng G, Yang N, Eltawil MA, Kamal M, Ali A, Kabeel AE (2016). A hybrid desalination system using humidification-dehumidification and solar stills integrated with evacuated solar water heater. Energy Convers. Manag..

[21] Su J, Zhang P, Yang R, Wang B, Zhao H, Wang W, Wang C (2022). MXene-based fl exible and washable photothermal fabrics for ef fi ciently continuous solar-driven evaporation and desalination of seawater. Renew. Energy.

[22] Choong WS, Ho ZY, Bahar R (2020). Solar desalination using fresnel lens as concentrated solar power device: An experimental study in tropical climate. Front. Energy Res..

[23] Abdelaziz GB, El-said EMS, Bedair AG, Sharshir SW, Kabeel AE, Mimi A (2021). Experimental study of activated carbon as a porous absorber in solar desalination with environmental, exergy, and economic analysis. Process Saf. Environ. Prot..

[24] Nijmeh S, Odeh S, Akash B (2005). Experimental and theoretical study of a single-basin solar still in Jordan. Int. Commun. Heat Mass Transf..

[25] Sharshir SW, Kabeel AE, Elsheikh AH, Peng G (2017). Thermal performance and exergy analysis of solar stills: A review. Renew. Sustain. Energy Rev..

[26] Yousef MS, Hassan H, Ahmed M, Ookawara S (2017). Energy and exergy analysis of single slope passive solar still under Egyptian climate conditions. Energy Proc..

[27] Zanganeh P, Soltani A, Ayatollahi S, Feilizadeh M (2020). Efficiency improvement of solar stills through wettability alteration of the condensation surface: An experimental study. Appl. Energy.

[28] Zanganeh P, Soltani A, Ayatollahi S (2020). Nano-coated condensation surfaces enhanced the productivity of the single-slope solar still by changing the condensation mechanism. J. Clean. Prod..

[29] Shoeibi S, Saemian M, Kargarsharifabad H, Hosseinzade S (2022). A review on evaporation improvement of solar still desalination using porous material. Int. Commun. Heat Mass Transf..

[30] Dhivagar R, Shoeibi S, Kargarsharifabad H, Ahmadi MH, Sharifpur M (2022). Performance enhancement of a solar still using magnetic powder as an energy storage medium—exergy and environmental analysis. Energy Sci. Eng..

[31] Modi KV, Modi JG (2019). Performance of single-slope double-basin solar stills with small pile of wick materials. Appl. Therm. Eng..

[32] Kabeel AE, Harby K, Abdelgaied M, Eisa A (2021). Performance improvement of a tubular solar still using V-corrugated absorber with wick materials: Numerical and experimental investigations. Sol. Energy.

[33] Saravanan A, Murugan M (2020). Performance evaluation of square pyramid solar still with various vertical wick materials: An experimental approach. Therm. Sci. Eng. Prog..

[34] Murugavel KK, Srithar K (2011). Performance study on basin type double slope solar still with different wick materials and minimum mass of water. Renew. Energy.

[35] Elgendi M, Kabeel AE, Essa FA (2023). Improving the solar still productivity using thermoelectric materials: A review. Alex. Eng. J..

[36] Peng G, Sharshir SW (2023). Progress and performance of multi-stage solar still: A review. Desalination.

[37] Singh, S., Tyagi, S. K. & Kaushik, S. C. Desalination using waste heat recovery with active solar still. In *Proceedings of the 7th International Conference on Advances in Energy Research* 439–447 (2021).

[38] Kumar A, Sathyamurthy R, Sharshir SW, Elnaby A, Shamsuddin M, Hwang J (2021). A novel reduced graphene oxide based absorber for augmenting the water yield and thermal performance of solar desalination unit. Mater. Lett..

[39] Guan W, Guo Y, Yu G (2021). Carbon materials for solar water evaporation and desalination. Small.

[40] Onofrei E, Rocha AM, Catarino A (2011). The influence of knitted fabrics’ structure on the thermal and moisture management properties. J. Eng. Fibres Fabr..

[41] Supuren G, Oglakcioglu N, Ozdil N, Marmarali A (2011). Moisture management and thermal absorptivity properties of double-face knitted fabrics. Text. Res. J..

[42] Senthilkumar M, Sampath MB, Ramachandran T (2013). Moisture management in an active sportswear: Techniques and evaluation—A review article. J. Inst. Eng. Ser. E.

[43] Sharma N, Kumar P, Bhatia D, Sinha SK (2016). Moisture management behaviour of knitted fabric from structurally modified ring and vortex spun yarn. J. Inst. Eng. Ser. E.

[44] Hussain, S., Glombikova, V., Havelka, A., Jamshaid, H., Batool, S. S. & Khan, M. Z. Moisture transport phenomena of functional underwears. *Vlákna a Text.* 59–66, (2017).

[45] Yanılmaz M, Kalaoğlu F (2012). Investigation of wicking, wetting and drying properties of acrylic knitted fabrics. Text. Res. J..

[46] Ozdil, N., Süpüren, G., Ozçelik, G. & Pruchova, J. A study on the moisture transport properties of the cotton knitted fabrics in single jersey structure. *Tekst. ve Konfeksiyon* 218–223 (2009).

[47] Fangueiro R, Filgueiras A, Soutinho F, Meidi X (2010). Wicking behavior and drying capability of functional knitted fabrics. Text. Res. J..

[48] Foshee, J. V. Knitted fabric with dual layer construction and method for making. US 7,360,378 B2 (2008).

[49] Furferi R (2023). A step-by-step method for predicting the spectrophotometric response of a carded fabric composed by differently colored raw materials. MethodsX.

[50] Moula ATMG, Hosen D, Bakar A, Momin A, Kaisar Z, Al A, Islam A (2022). Effect of dye bath pH in dyeing of cotton knitted fabric with reactive dye (Remazol Yellow RR) in exhaust method: Impact on color strength, chromatic values and fastness properties. Heliyon.

[51] Shahid M, Ali A, Zahid N, Anjam MS, Militky J, Wiener J, Palanisamy S, Tomkova B (2023). Copper-treated environmentally friendly antipathogenic cotton fabric with modified reactive blue 4 dye to improve its antibacterial and aesthetic properties. Coatings.

[52] Elshamy SM, El-said EMS (2018). Comparative study based on thermal, exergetic and economic analyses of a tubular solar still with semi-circular corrugated absorber Total Cost of Ownership. J. Clean. Prod..

[53] Kabeel AE (2009). Performance of solar still with a concave wick evaporation surface. Energy.

[54] Masoud S, Norouzpour F, Shoeibi S, Shahsavar A (2023). A comprehensive study to find the optimal fraction of nanoparticle coated at the interface of solar desalination absorbers: 5E and GHGs analysis in different seasons. Sol. Energy Mater. Sol. Cells.

[55] Dhivagar R, Shoeibi S, Masoud S, Hoseinzadeh S, Kargarsharifabad H, Khiadani M (2023). Performance evaluation of solar still using energy storage biomaterial with porous surface: An experimental study and environmental analysis. Renew. Energy.

[56] Masoud S, Yazdani A, Javadi D, Afrand M, Karimi N, Muhammad H, Yves J (2022). Selecting efficient side of thermoelectric in pyramid-shape solar desalination units incorporated phase change material (PCM), nanoparticle, turbulator with battery storage powered by photovoltaic. J. Energy Storage.

[57] Rashidi S, Rahbar N, Sadegh M, Abolfazli J (2018). Enhancement of solar still by reticular porous media: Experimental investigation with exergy and economic analysis. Appl. Therm. Eng..

[58] Ghani H, Azeez H, Diabil N, Al-moussawi MA (2023). A numerical investigation of the enhancement of single-slope single-basin solar still productivity. Energy Rep..

[59] Al-Shabibi, A. M., & Tahat, M. Thermal performance of a single slope solar water still with enhanced solar heating system key words. In *International Conference on Renewable Energies and Power Quality* vol. 1, no. 13, 585–587 (2015).

[60] Shoeibi S, Ali S, Mirjalily A, Kargarsharifabad H, Panchal H, Dhivagar R (2022). Comparative study of double—slope solar still, hemispherical solar still, and tubular solar still using Al_2_O_3_/water film cooling: A numerical study and CO_2_ mitigation analysis. Environ. Sci. Pollut. Res..

[61] Pal P, Kumar S, Bharti A, Narayan A, Dev R (2021). Energy, exergy, energy matrices, exergoeconomic and enviroeconomic assessment of modified solar stills. Sustain. Energy Technol. Assess..

[62] Kabeel AE, Abou T, El-said EMS (2013). Economic analysis of a small-scale hybrid air HDH e SSF (humidi fi cation and dehumidi fi cation e water fl ashing evaporation) desalination plant. Energy.

[63] Shoeibi S, Kargarsharifabad H, Ali S, Mirjalily A, Muhammad T (2022). Solar district heating with solar desalination using energy storage material for domestic hot water and drinking water: Environmental and economic analysis. Sustain. Energy Technol. Assess.

[64] Mohamed AF, Hegazi AA, Sultan GI, El-said EMS (2019). Enhancement of a solar still performance by inclusion the basalt stones as a porous sensible absorber: Experimental study and thermo-economic analysis. Sol. Energy Mater. Sol. Cells.

[65] Mohanraj RDM, Ye KH (2021). Energy, exergy, economic and enviro-economic (4E) analysis of gravel coarse aggregate sensible heat storage-assisted single-slope solar still Bureau of Indian Standards. J. Therm. Anal. Calorim..

[66] Shoeibi S, Rahbar N, Abedini A, Kargarsharifabad H (2021). Energy matrices, exergoeconomic and enviroeconomic analysis of air- cooled and water-cooled solar still: Experimental investigation and numerical simulation. Renew. Energy.

[67] El-Said EMS, Elshamy SM, Kabeel AE (2020). Performance enhancement of a tubular solar still by utilizing wire mesh packing under harmonic motion Water trough. Desalination.

